# The Family

**Published:** 1866-11

**Authors:** Joel Parker


### THE FAMILY.

BY REV. JOEL PARKER, D. D.

THE Family is a wondrous thing. What a history it has! It dates clear back to Eden. It commences with the creation of the first man. In a sense, it is all contained in him, as its germ; for the woman and the offspring are a development from the primordial form. The family is a house built by an infinitely skillful Architect. It is commenced with one living stone, and thence "groweth unto an holy temple in the Lord." Adam is "the head of the corner"—a corner-stone, stretching out in one direction. The woman is developed from the man, and the two stand as two walls united by a right-angle. By a double birth, the twin brothers, Cain and Abel, become the complement of the square. There stands, in its most complete form, our human life, as the life of Christ is set forth by the four-sided revelation of the Evangelists. And yet, while there is a four-fold form in the first complete family, as indicating strength and progressiveness beyond the necessary elements, there is a threefoldness analogous to the Divine Subsistence in a primal source in the man, in a second one proceeding from him in woman, and in a procession from the first two united, in their children.

Satan's envy was first excited by beholding this faint emblem of the Holy Trinity in the three-foldness of man, woman, and offspring; and, as the arch tempter sought first to debase the image of God in the family, so we must commence a holy influence in that very spot, and first "bind the Devil on the hearth-stone." Call this philosophy, or speculation, or what you will, the family is a great institution. It is the germ of every valuable social organization—the State, the Church, the School, and the compact of combined labor.

The greatest function of the family, next to its physical subsistence, growth, and increase, which are essential to its existence, is intellectual and spiritual development. In this, all the members bear a part. Like the allegory of the body and its several limbs and members, by Menonius Agrippa, and like St. Paul's similitude, borrowed from it, and invested with the life and perfection of inspiration, the family has a head and heart, and hands and feet, and all the various functions of a body. The family is an embryo state—a government. Its form, it is true, is peculiar. It is not a despotic empire, nor a monarchy, nor a representative republic, nor a pure democracy. It is, certainly, a patriarchal government—a divinely organized theocracy. Thus, the family has a head, as God is the head of the moral universe. As the Lord said to Moses, "I have made thee a god to Pharaoh: and Aaron thy brother shall be thy prophet"—so, in effect, the Lord has made the father a god to his family; and the wife and mother is clad in a priestly stole, like Aaron, and is made the prophet of this divinely constituted organization.

As a Church, it possesses features and arrangements analogous with those of a fully developed Christianity. The husband is in the place of Christ; his wife is in the place of the Church; and the children ought to be a holy brotherhood. The ligaments that bind them all together, are those of love. The voice of worship, of prayer, and harmonious holy song, should fill the family mansion, and impart to the community that sacred character which justified an apostle, when sending greetings to a friend, in subjoining, after making mention of individually, "And to the church which is in thy house."

The family ought to be more earnestly employed, as an organization, for securing every valuable end of social existence. It ought to be catechised in the elements of sacred truth, inspired with holy sentiments, and directed in the employment of its practical powers. To this end, a loving unity is of the very highest consequence. Nothing is more conducive to this than family worship and inspired teaching. There is a single psalm, the 133d, which is, perhaps, the most beautiful, and the sweetest bouquet in the whole Hebrew anthology. Let it be made familiar to all, as if placed in a vase in the keeping-room, and its beauty and fragrance shall fill the place. "Behold, how good and how pleasant it is for brethren to dwell together in unity! It is like the precious ointment upon the head, that ran down upon the beard, even Aaron's beard; that went down to the skirts of his garments: As the dew of Hermon, and as the dew that descended upon the mountains of Zion; for there the Lord commanded the blessing, even life for evermore." All the members of a household ought to make this beautiful lyric their own.

Then let all take part in the work of educating the family. What a beautiful, and useful, and pleasure-producing employment is it, to read in the family-circle! Let there be stated times for the exercise. Let every one select gems of thought—elegant literature, scientific truth, rare and interesting information, and bring it forward for the general good. A good article is as quickly read as a poor one. A single paragraph, of extraordinary force and beauty, is of more value than whole volumes of fair common-place reading. One scientific truth, clearly brought out, will furnish matter for useful conversation, and reflection, and profitable application, for many years to come.

Let the individual members be appointed each to seek out and present some specific needed information. Appoint each one to lecture on a given subject. Give to one, for instance, "printing," as a theme, with instructions to acquire and present to the family-circle, in a compact form, information on the whole subject, embracing the etymology of the word *to print;* the origin of the art—its progress—its present extent in the world—its connection with other discoveries and improvements, as paper and ink manufacture, and printing-presses, and types, stereotyping and electrotyping, and their present and prospective influence upon the world. Give to another, Africa, embracing its physical geography, and races, and languages, with commerce, and productions, and travels, and colonization, and present and prospective civilization—slavery, soil, climate, and indeed everything that can awaken an intelligent interest. Let another prepare a lecture on chemical science; another, on the beautiful; another, on the vast; and another, on the minute. Give time for preparation by reading and inquiry. Allow months or weeks—if needs be, a year. To this lyceum work, manufacture may be added; and every member may be required to produce something distinguished for elegance or use. Let song, and utterance, and the vernacular tongue, be cultivated. Let sketches of excursions be preserved, and scraps of great value be clipped from periodical literature, and preserved. These are mere hints. Carry the purpose of improvement out in all directions. Make the family a university.

---

Parental Responsibility.—The father molds the head; the mother, the heart; the father appeals to the understanding; the mother, to the affections; the father prepares for time; the mother, for eternity. Happy the children who heed the wise teachings of both.

